# Use Of Appendix As Neoureter- A Ray Of Hope

**DOI:** 10.21699/jns.v6i3.576

**Published:** 2017-08-10

**Authors:** Parveen Kumar, Yogesh Kumar Sarin

**Affiliations:** Department of Pediatric Surgery, MAMC, New Delhi

**Keywords:** PUJO, Neoureter, Ureteric replacement, Appendix

## Abstract

A case of antenatally diagnosed hydronephrosis (later known to be PUJO complicated with urinoma and associated with hypoplasia of entire ureter) was treated using vermiform appendix as replacement.

## INTRODUCTION

In today’s era of reconstructive surgeries, appendix is no more considered a vestigial structure. Its use as Mitrofanoff and Split Appendix in Malone’s Antegrade Continent Enema procedure are well time-tested. Here, we present another use of appendix.

## CASE REPORT

A male neonate born at full-term through normal vaginal delivery was brought for consultation for a prenatal diagnosis of right hydronephrosis (3rd Trimester scan APPD 4.2 cm). Baby was born with distended abdomen and respiratory distress. Examination revealed tachypnea (RR =78/min), HR 138/min, CFT <3sec, Temp = 36.8 C0. Abdomen was grossly distended, tense, dull on percussion and bowel sounds were absent. Hemoglobin was 19.7 g %, TLC 16900, platelet 2.4 lakhs, blood urea 24 mg%, creatinine 0.8 mg%, Na 138 and K 5.3 meq/l. Infantogram showed cardiomegaly and soft-tissue shadow occupying most of the right half of the abdomen; bowel loops were shifted to left. 2D-ECHO revealed Atrial septal defect with dilated right atrium and ventricle. Ultrasound showed non-visualization of right kidney and presence of large thick walled cystic structure in right renal fossa. Baby underwent exploratory laparotomy with drainage of right retroperitoneal urinoma and right Anderson Hyne’s reduction pyeloplasty. Post-operative period was uneventful. On POD -10, a right nephrostogram was done, which showed blocked anastomosis. At 1 month of age, diuretic renography (DR) showed 16 % DRF(Differential Renal Function) of right kidney. The baby underwent re-exploration and the hypoplastic ureter was replaced by appendix and brought out as end right ureterostomy (Fig.1). At the age of 3 months, the baby had pyuria (E.coli) from right end ureterostomy; the neo-ureter made out of appendix was catheterized and culture-sensitive antibiotics were administered and patient responded well.

**Figure:**
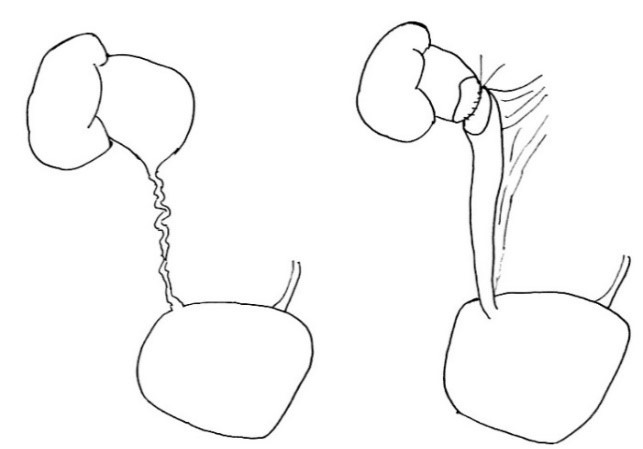
Figure 1: PUJ. Use of appendix to replace ureter.

At 1 year of age, DR showed DRF 21.5% and GFR 22.4 ml/min for right kidney. Endoscopy via appendiceal lumen (neoureter), followed by implantation of distal appendix end to bladder using extra-vesical Leisch-Gregor technique, with DJ stenting was done. Eight weeks postoperative, DJ stent was removed. DR done at 6 month post last surgery showed some deterioration of function (DRF 15 % and GFR of 7.5 ml/min). At last follow-up 1.5 years post-surgery, DRF right kidney was 13.3% and GFR of 10.1 ml/min, with child being asymptomatic.

## DISCUSSION

The goal of surgery for PUJO is to preserve renal function by assisting unobstructed drainage of the renal pelvis. This technique of partial or total replacement of ureter by appendix has the potential of being a technically feasible option in long segment ureteric stenosis/necrosis. [1,2,3]

To conclude, appendix is a potential conduit for replacement of whole or partial ureter helping renal salvageability, though larger series and longer follow up needed to lay consensus.

## Footnotes

**Source of Support:**None

**Conflict of Interest:** None

## References

[1] Dagash H, Sen S, Chacko J, Karl S, Ghosh D, Parag P, Mackinnon AE. The appendix as ureteral substitute: a report of 10 cases. J Pediatr Urol. 2008; 4:14-9. 10.1016/j.jpurol.2007.08.004 18631886

[2] Deyl RT, Averbeck MA, Almeida GL, Pioner GT, Souto CA. Appendix interposition for total left ureteral reconstruction. J Pediatr Urol. 2009;5:237-9. 10.1016/j.jpurol.2008.11.010 19109072

[3] Obaidah A1, Mane SB, Dhende NP, Acharya H, Goel N, Thakur AA et al. Our experience of ureteral substitution in pediatric age group. J Urology. 2010; 75:1476-80. 10.1016/j.urology.2009.07.1327 19913889

